# Concrete Aggregate-Gradation Effect and Strength-Criterion Modification for Fully Graded Hydraulic Concrete

**DOI:** 10.3390/ma17153816

**Published:** 2024-08-02

**Authors:** Chao Wang, Qingming Qiu, Xiaohua Wang, Sherong Zhang, Gaohui Wang, Peiyong Wei

**Affiliations:** 1 State Key Laboratory of Water Resources Engineering and Management, Wuhan University, Wuhan 430072, China; wanggaohui@whu.edu.cn; 2 State Key Laboratory of Hydraulic Engineering Simulation and Safety & School of Civil Engineering, Tianjin University, Tianjin 300072, China; wangchaosg@tju.edu.cn (C.W.); lemoncc717@126.com (Q.Q.); tjuzsr@126.com (S.Z.); wepy@tju.tdu.cn (P.W.)

**Keywords:** fully graded concrete, aggregate gradation, strength criterion, mesoscopic numerical simulation, triaxial compressive test

## Abstract

Utilization of large aggregates can promote energy conservation and emissions reductions, and large aggregates have been widely used in hydraulic concrete. The failure criterion for concrete material utilizing large aggregates forms the basis for constitutive models and structural design. However, the concrete failure criterion with respect to large aggregates has never been researched. To this end, the authors first conducted a series of triaxial compressive tests on concrete specimens with scaled aggregates. On this basis, several 3D mesoscopic numerical models were established with different aggregate gradations and used to simulate the triaxial compressive behaviors of hydraulic concrete after the models had been verified by experimental results. The results showed a pronounced aggregate-gradation effect on triaxial compressive behaviors, and concrete mixes with larger aggregates usually have higher compressive strength, especially under conditions of higher confinement. The normalized peak strength can increase by up to 23.49%. Finally, based on the available testing data, the strength criterion in different constitutive models is discussed and modified to allow more accurate simulation of the dynamic responses of and damage to fully graded concrete structures. This result can provide a theoretical basis on which construction entities can optimize the mix proportions of fully graded concrete and detect the failure modes of concrete structures.

## 1. Introduction

The aggregate in concrete usually constitutes 70–80% of the concrete by volume and is considered to be inert filler. As the technology of concrete advances, it has been recognized that both the size and the type of the aggregate have significant and decisive influences on various important properties of concrete, such as compressive and tensile strength, volume stability, and durability [1]. Aggregate in concrete also shows a significant size-related sensitivity to many physical and mechanical properties, and it has been verified that hydraulic concrete with a maximum aggregate size reaching 120 mm usually has low porosity, a small interfacial transition zone, and low water and cement consumption [2,3].

Aggregate gradation or maximum aggregate-particle size usually has a significant effect on many important characteristics of concrete. It has been concluded that the contributions from aggregates to the dynamic mechanical behaviors of concrete are considerable, especially at high loading rates. The presence of coarse aggregates can enhance the dynamic increase factors (DIFs) of specimens. The material heterogeneity increases, whereas the obtained DIFs decreases and become more dispersed, as the maximum aggregate-particle size increases. Also, increasing the aggregate content in concrete can enhance the dynamic compressive strength of concrete materials at high loading rates [1,4,5]. Recently, researchers have paid attention to the effect of concrete aggregate size, but progress is relatively slow due to equipment-size limitations that make experiments very difficult to conduct. Therefore, coarse aggregates with a maximum size of 20 mm are commonly used in laboratory concrete tests to meet the uniformity requirement of specimens, ignoring the aggregate’s effect on the mechanical behaviors of concrete [6,7,8]. By contrast, large aggregates are often used in practical engineering construction, and concrete mix designs of grade three (maximum aggregate size *d*_max_ = 80 mm) and grade four (*d*_max_ = 150 mm) are selected for most hydraulic dams. Therefore, the results of mechanical tests conducted in the laboratory on concrete with small aggregate gradations cannot capture the mechanical behaviors of fully graded concrete because of the aggregate-gradation effect [9].

The constitutive relationship is a necessary basis for structural analysis, and the concrete strength criterion is the key element of the concrete constitutive model, which describes the peak strength under various loading conditions [10]. The microcracks inside the concrete continue to expand under the action of triaxial compression and eventually develop into visible cracks, finally leading to failure of the material. This progressive failure process can be generalized using the concrete plastic constitutive model, and the failure criteria of normal concrete have been extensively studied by many researchers using various methods [11,12]. However, for hydraulic concrete with large aggregates, its failure criterion is not well understood; current data stress influence of the specific aggregate gradation on behaviors. The limited existing experimental investigations have verified that the lateral stress can enhance the strength of hydraulic concrete, yet the explicit model used to forecast the behaviors of hydraulic concrete covering the aggregate-gradation effect under triaxial stress conditions has not been published [13,14]. In an investigation of the aggregate-gradation effect of hydraulic concrete, mesoscopic numerical simulation provides a convenient and reasonable approach by which to study the failure mechanism and establish relationships between the mesofracture and macromechanical behaviors of concrete-like materials [15,16,17]. A mechanical test based on the mesoscopic numerical model can effectively avoid the problems of high laboratory-test costs, equipment-size limitations and large measurement errors. In addition, in order to provide the basis for the modification of the concrete constitutive model, it is necessary to analyze the influence of heterogeneous mesostructure on the macroscopic static and dynamic behaviors of concrete.

In concrete mesomechanics, the inner mesostructure of concrete can be treated as a multiphase composite structure consisting of coarse aggregate, mortar, and an interfacial transition zone (ITZ). To this end, Tian [18] established a two-dimensional mesoscale numerical model of concrete with different aggregate gradations, including grade two (5 mm~40 mm), grade three (5 mm~80 mm) and grade four (5 mm~150 mm), so as to take aggregate-gradation effect into consideration, and it was shown that aggregate gradation and particle size have a significant influence on the compressive behaviors of concrete materials. Zhou et al. [19] combined laser scanning with spatial cutting technology to establish a dataset of real aggregate shapes and conducted a deep investigation into the influence of aggregate shape and size on the uniaxial compressive strength of concrete. However, the concrete aggregate-gradation effect can be studied using three-dimensional mesonumerical models. For example, the effects of specimen size, aggregate gradation, and aggregate content on the splitting tensile strength of concrete at various loading rates were considered, and it was verified that the splitting tensile strength increased as the aggregate size and content increased, but the specimen-size effect at high loading rates was not obvious [20]. Jin et al. [21] discussed the influences of aggregate size and specimen grade on the failure behavior at low strain rates based on mesosimulations, and the results verified that the concrete tensile strength increased with increasing aggregate size and that the compressive strength first increased but then decreased with increasing aggregate size.

This study focuses on developing a deeper understanding of the aggregate-gradation effect on concrete and then on calibrating the existing strength criterion for fully graded hydraulic concrete with due consideration of the aggregate gradation. A series of three-dimensional mesoscopic numerical models was first established and then verified using experimental results, and then the triaxial compressive behaviors of hydraulic concrete with different aggregate gradations are investigated by mesoscopic numerical simulation. Finally, the different existing failure criteria developed based on normal concrete are discussed in terms of their performance and the criteria are modified to reflect the aggregate-gradation effect on the failure surfaces of fully graded concrete.

## 2. Experimental Triaxial Compressive Behaviors for Scaled Hydraulic Concrete

### 2.1. Test 

**Figure 1:**
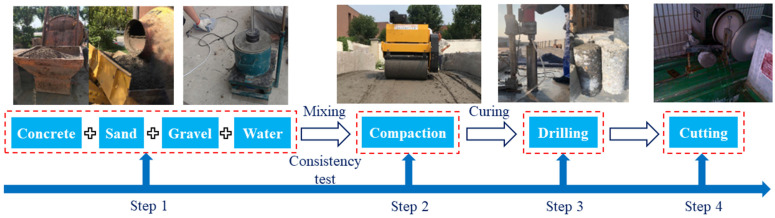
Specimen-preparation process.

Considering the influence of mix design and layered structure on the mechanical behaviors of hydraulic concrete, a specimen-preparation procedure of vibration and compaction similar to that used in field concrete construction was conducted. The material-preparation and specimen-production procedures have been explained in our previous studies in detail [8,13]. It is noted that the aggregates used in specimen preparation were scaled to 20 mm so as to adapt to the size of triaxial compressive equipment; the product was termed “scaled hydraulic concrete” in this study. First, cement (Tianjin Shanshui Cement Co., Tianjin, China), sand, stone, and water were mixed, and the VC value of the concrete was tested. After that, the concrete was crushed and cured. Then, a set of 100 mm hydraulic concrete cores were drilled and these cores were cut and ground for both uniaxial and triaxial compressive tests. The specific process is shown in Figure 1. During the tests, axial displacement and circumferential deformation were measured using an axial extension meter and a circumferential extension meter. By these means, the deformation along the entire circumference of the cylindrical specimen can be monitored and the average lateral strain can be derived from this deformation. In both uniaxial and triaxial compressive tests, a displacement-control mode at a set loading rate of 0.002 mm/s was used.

**Figure 2:**
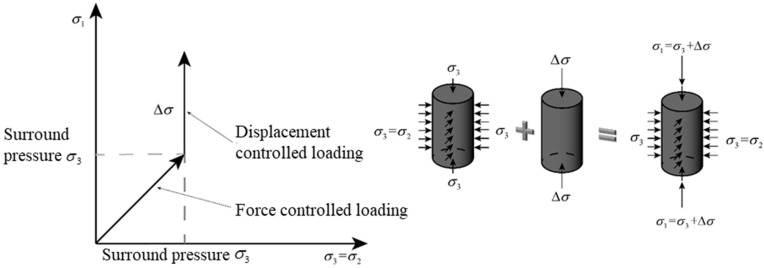
Schematic diagram of triaxial test loading path.

For triaxial compression, the load path is illustrated in Figure 2. The confinement was first applied to the specimen and increased to the target confinement ($\sigma_{3}$) in load-control mode. Then, deviatoric load was applied in the displacement-control mode (set 0.002 mm/s loading rate), until specimen failure. A total of seven confinements were tested, i.e., 0 MPa, 5 MPa, 10 MPa, 15 MPa, 20 MPa, 25 MPa, and 30 MPa, with the corresponding confinement ratio ($\sigma_{3}/f_{c}$) varying from 0.00 to 2.01. To prevent the confining fluid from penetrating into the concrete during the test, it is necessary to cover the hydraulic concrete specimen with a 1.5 mm thick heat-shrink tube. At each confining pressure level, the stress–strain curves were averaged from three reasonable test results.

### 2.2. Test Results

**Figure 3:**
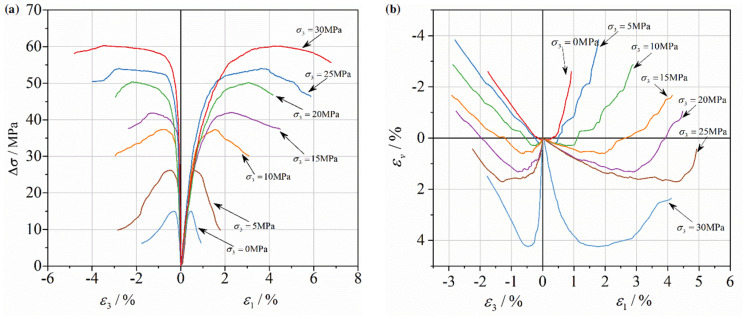
Triaxial compressive behaviors of hydraulic concrete under different confinements: (**a**) deviatoric stress–strain curves; (**b**) volumetric–strain–time history curve.

Figure 3 describes the histories of deviational stress and volumetric strain for hydraulic concrete at different confinements during the triaxial tests. The definitions of axial stress $\sigma_{1}$ and volumetric strain $\varepsilon_{v}$ can be illustrated as Equations (1) and (2). It is clear that the deviatoric stress ($\Delta \sigma =\sigma_{1}-\sigma_{3}$) increases significantly as the confinement increases at a given loading rate. Furthermore, the hydraulic concrete specimen exhibits more obvious ductile and plastic behavior at a higher confinement level. Meanwhile, lateral dilation occurred, accompanied by axial compression due to Poisson’s law. Crack generation and propagation occur in this process under the confinement restriction.

(1) $$\begin{array}{c} \sigma_{1}=\Delta \sigma +\sigma_{3}=F_{N}/A_{s}+\sigma_{3} \end{array}$$

(2) $$\begin{array}{c} \varepsilon_{v}=\varepsilon_{1}-2\varepsilon_{3} \end{array}$$

where $\sigma_{1}$ and $\sigma_{3}$ denote the axial stress and confinement level of specimen, respectively; Δσ represents the deviatoric stress; and $\varepsilon_{v}$, $\varepsilon_{1}$ and $\varepsilon_{3}$ are the volumetric strain, axial strain and radial strain, respectively.

In addition, softening behavior of concrete specimens can be observed in Figure 3 under all kinds of confinement levels (0 MPa–30 MPa). It is obvious that the peak strength and the corresponding axial strain get higher as the confinement increases. The volumetric strain tends from contraction to dilation as the cracks develop. In addition, the concrete specimens show more plastic behavior under relatively higher confinements (15 MPa–30 MPa). The post-peak curves decrease in steepness as confinement increases, finally reaching a stable state. The formula is as follows:

(3) $$\begin{array}{c} \sigma_{1}/f_{c}=1+k\sigma_{3}/f_{c} \end{array}$$

in which k=(1+sinφ)/(1−sinφ) and φ is the internal-friction angle.

**Figure 4:**
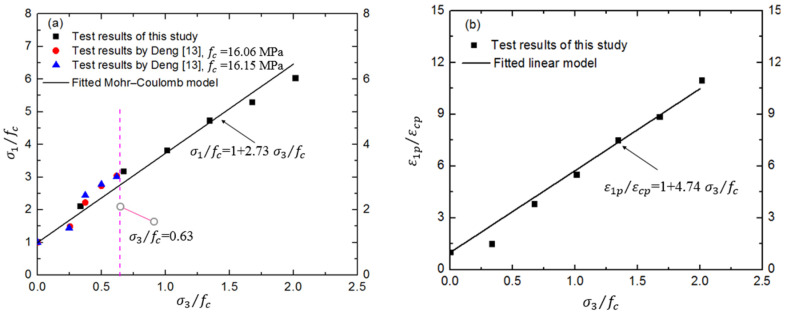
Peak strength and corresponding strain at different confinements. (**a**) Relationship between peak strain and confinement; (**b**) Relationship between ultimate strain and confinement [13], where $\varepsilon_{cp}$ is the peak axial strain of concrete under uniaxial compression.

Figure 4 shows the experimental data for the scaled hydraulic concrete specimens in triaxial compressive tests, compared with the available data from a real RCC dam. However, the maximum confinement ratio from the existing dam is limited to 0.63. Thus, this study further researches the triaxial compressive behaviors at higher confinements. It is clear in Figure 4 that both the normalized axial compressive strength and corresponding strain get higher as the confinement increases, showing a nearly linear relationship between them. The Mohr–Coulomb failure criterion expressed by Equation (3) was used in this study to simply illustrate the strength surface, as shown in Figure 4. More discussion of the strength criterion is conducted in Section 4, below.

## 3. Numerical Investigation of Triaxial Compressive Behaviors for Hydraulic Concrete

### 3.1. Establishing and Verification of Mesoscopic Numerical Model

To describe the triaxial compression behavior of fully graded hydraulic concrete under high confining pressure more accurately, this subsection applies three-dimensional mesoscopic numerical simulation to further consider the aggregate-gradation effect on the triaxial compressive behavior of hydraulic concrete. In the mesoscopic numerical models, three components, namely, mortar matrix, coarse aggregate, and ITZ, are considered. Mortar mainly composed of fine aggregate ($d_{max}<5\;mm$) and cement slurry is usually generalized into uniform and isotropic material in mesonumerical simulations. As for the coarse aggregate, its lithology, shape, stiffness, content and gradation are proved to impact the macroscopic mechanical behaviors of concrete at different levels. The ITZ around the coarse aggregates is usually about 20 μm~50 μm [22,23].

**Figure 5:**
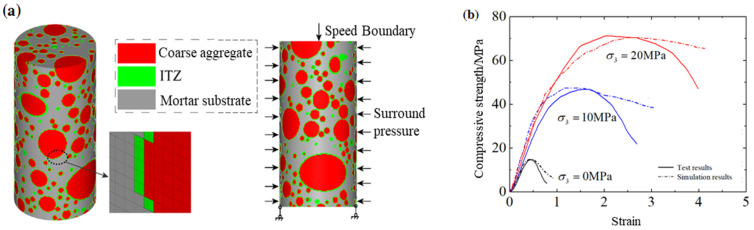
Triaxial compressive tests by mesosimulation: (**a**) mesoscopic numerical model and loading schematic; (**b**) model verification.

Due to its high porosity and mesocracks, ITZ is often considered to be the weakest link in concrete. Considering the calculation efficiency and accuracy, the mesoscopic numerical models built in this study set the ITZ thickness within the range of 0.5 mm~2.0 mm [22,23], and the random defects are not considered in this study, as has been done in several previous articles in the literature [22,23]. Figure 5a shows the mesoscopic numerical model with an aggregate gradation of 5 mm~20 mm that was built to simulate the above triaxial compressive tests on scaled hydraulic concrete, in which spherical coarse aggregates of 5 mm~20 mm were randomly distributed in the numerical model.

In this study, the KCC constitutive model was used to simulate the behavior of these three mesoscopic components and the parameter automation generation in LS-DYNA was used to generate parameters for different components. The only necessary input parameters are the density, uniaxial compressive and tensile strengths, Poison’s ratio, and shear modulus. Since it is difficult to determine the model parameters of ITZ, the ITZ is generalized as a material weakening of mortar matrix. Table 1 lists the model input parameters for a KCC model of mesoscopic concrete components. Then, triaxial compression was conducted on the numerical model according to the loading path in Figure 1. The model boundaries are illustrated by Figure 5a. The mesosimulation results are compared with those of laboratory tests in Figure 5b. It has been confirmed that the stress–strain curves under different confinements from the numerical simulation are consistent with those from laboratory tests. That means the mesoscopic numerical model and corresponding parameters are a reliable means by which to further study the aggregate-gradation effect on the triaxial compressive behaviors and strength criterion of hydraulic concrete, although there exists an obvious gap in the softening behavior due to rough parameter selection.

### 3.2. Triaxial Compressive Behaviors of Different Grades of Hydraulic Concrete 

By the verified mesosimulation method, another three kinds of numerical model with aggregate gradations of 5 mm~40 mm, 5 mm~60 mm, and 5 mm~80 mm were built to study the triaxial compressive behaviors of hydraulic concrete. Seven confinement ratios, i.e., $\sigma_{3}/f_{c}$ = 0.00, 0.34, 0.67, 1.01, 1.34, 1.68, and 2.01, were used to reveal the relationship among aggregate gradation, peak compressive strength, and confinement level. The numerical results are listed in Table 2, where they are compared with the results of the laboratory tests conducted in Section 2.

It is obvious that the numerical compressive strengths are consistent with those from laboratory tests at various confinement levels. Moreover, the aggregate-gradation effect on concrete compressive strength is inapparent at low confinements but becomes more obvious with increasing confining pressure. Moreover, the aggregate-gradation effect is more obvious for concrete with larger aggregates. For example, when the maximum aggregate increases from 20 mm to 80 mm, the increase in normalized peak strength reaches 23.49% at a confinement ratio of 2.01. The reason may be that smaller coarse aggregates lead to more ITZs; the confining pressure can further amplify the effect of ITZs, resulting in attenuation of concrete strength under triaxial compression. Thus, the aggregate-gradation effect of hydraulic concrete will have a significant influence on dynamic behaviors under confinements such as extreme blast and impact loads. The formula is as follows:

(4) $$\begin{array}{c} \sigma_{1}/f_{c}=1+2.635(\sigma_{3}/f_{c}){\left(d_{max}/d_{0}\right)}^{0.161} \end{array}$$

where $d_{0}$ is referenced aggregate size and equals 20 mm; $d_{max}$ represents the maximum aggregate size in the concrete mix design.

**Figure 6:**
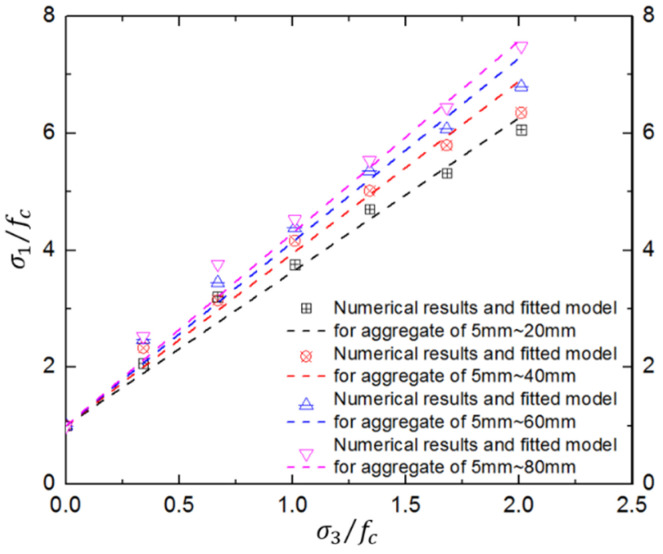
Results of triaxial compression tests on concrete with different aggregate gradations.

In order to further describe the aggregate-gradation effect, an aggregate-gradation term was introduced into the Mohr–Coulomb strength criterion, and the parameters are fitted by the numerical results in Table 2 as shown in Equation (4) and Figure 6. It was found that the modified Mohr–Coulomb strength criterion can effectively describe the general aggregate-gradation effect on the concrete strength surface. The aggregate-gradation effect becomes more significant at a higher confinement level. However, it is also clear that the modified Mohr–Coulomb strength criterion will overestimate the failure strength and that the strength surface is not linear at the high confinements. Thus, it is necessary to discuss the strength criterion in depth.

## 4. Discussion of the Strength Criterion of Fully Graded Concrete

### 4.1. Background of Concrete Strength Criterion

In order to investigate the strength criterion of fully graded concrete, datasets from triaxial compressive tests are needed for determination of the strength surfaces. However, the triaxial compressive behaviors of hydraulic concrete under various confinement levels have seldom been studied, and the only available data in the literature are described as follows. Deng drilled specimens from an existing dam and performed a series of triaxial compressive tests, in which the maximum confining pressure and confinement ratios were 10 MPa and 0.62, respectively [14]. The results showed that hydraulic concrete usually has a higher elasticity but a relatively lower triaxial compressive strength compared to normal concrete. Zhang et al. also conducted a series of triaxial compressive tests on laboratory hydraulic concrete specimens with the maximum aggregate size of 20 mm, and the maximum confinement reached 30 MPa [13]. Here, numerical simulation was used to study the triaxial compressive behaviors of hydraulic concrete with different aggregate gradations and the strength criterion was modified using the available experimental data and numerical results.

**Figure 7:**
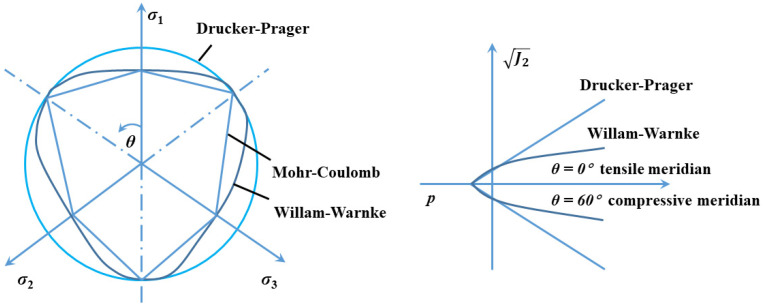
Strength surfaces in the deviatoric stress space and meridian plane.

For a certain material, the strength surfaces can be illustrated by the three-dimensional stress space, and Figure 7 and Equation (5) describe the stress-space transformation from the Haigh–Westergaard stress space (principal stresses, $\sigma_{3}\geq \sigma_{2}\geq \sigma_{1}$) to the cylindrical stress space ($p,\sqrt{J_{2}},\;\theta$). Then, in a traditional triaxial loading test, cylindrical specimens are confined by hydrostatic pressure within the stress state of $\sigma_{2}=\sigma_{3}$ and θ=60°, while θ varies within the range of 0° to 60° in true triaxial loading test. Thus, the strength surfaces of concrete can be described by the compressive meridian (θ=60°), the tensile meridian (θ=0°), and a curve connecting the two meridians (0°<θ<60°) in the deviatoric plane. The formula is as follows:

(5) $$\begin{array}{c} \left\{\begin{array}{c} p=\frac{1}{3}\left(\sigma_{1}+\sigma_{2}+\sigma_{3}\right)\\ \sqrt{J_{2}}=\frac{1}{\sqrt{6}}\sqrt{{\left(\sigma_{1}-\sigma_{2}\right)}^{2}+{\left(\sigma_{2}-\sigma_{3}\right)}^{2}+{\left(\sigma_{1}-\sigma_{3}\right)}^{2}}\\ \cos\theta =\frac{2\sigma_{1}-\sigma_{2}-\sigma_{3}}{\sqrt{2}\sqrt{{\left(\sigma_{1}-\sigma_{2}\right)}^{2}+{\left(\sigma_{2}-\sigma_{3}\right)}^{2}+{\left(\sigma_{1}-\sigma_{3}\right)}^{2}}} \end{array}\right. \end{array}$$

in which p, $\sqrt{J_{2}}$, and θ are coordinates in the cylindrical stress space and θ equals the Lode angle.

On this basis, scholars have proposed many strength-surface models of concrete-like materials, such as the Mohr–Coulomb criterion, Drucker–Prager criterion and Willam–Warnke criterion [11,12], as shown in Figure 7. Section 3.2 discusses the modification of the Mohr–Coulomb strength criterion and indicates that the aggregate-gradation effect can significantly influence the concrete failure surfaces at high confinements such as extreme blast and impact loads. Thus, the strength criteria in concrete dynamic constitutive models are further discussed below. For the concrete dynamic constitutive model, the Drucker–Prager criterion is used in the HJC model and the Willam-Warnke criterion is used in the RHT model and the KCC model.

### 4.2. Strength-Criterion Modification for the KCC Model

The strength criterion in the KCC model is described by three independent strength surfaces to illustrate the initial-yield strength surface $\sigma_{y}$, the maximum strength surface $\sigma_{m}$, and the residual strength surface $\sigma_{r}$, as illustrated in Equation (6), below:

(6) $$\begin{array}{c} \left\{\begin{array}{c} \sigma_{y}=a_{0y}+p/\left(a_{1y}+a_{2y}p\right)\\ \sigma_{m}=a_{0m}+p/\left(a_{1m}+a_{2m}p\right)\\ \sigma_{r}=a_{0r}+p/\left(a_{1r}+a_{2r}p\right) \end{array}\right. \end{array}$$

in which p represents the hydrostatic pressure; $a_{0i}$, $a_{1i}$ and $a_{2i}$ denote the parameters determining the strength surfaces, and i can be *y*, *m* or *r*.

**Figure 8:**
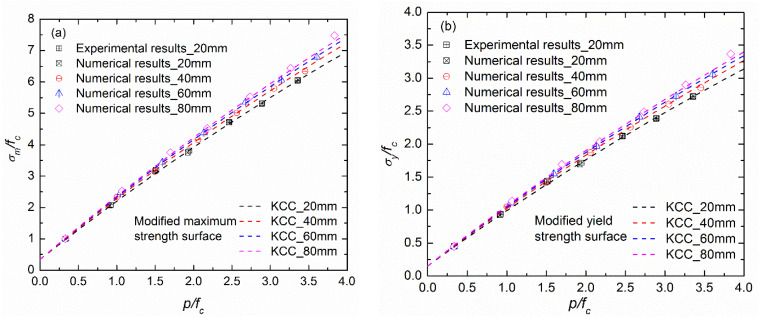
Determinations of modified strength criterion in the KCC model for fully graded concrete: (**a**) normalized maximum strength surface; (**b**) normalized initial-yield strength surface.

Figure 8 compares the testing data and the numerical results in this study with due consideration of the aggerate gradation; the normalized pressure and normalized deviator stress are used by dividing by the unconfined quasi-static strength of each group. Also, the yield strength, defined as 45% of the compressive strength, is also compared in Figure 8 [24]. It is obvious that both the maximum strength and the yield strength of concrete are sensitive to the aggregate gradation, although this aggregate-size effect is inapparent at low confinements ($p/f_{c}<1.5$). On the other hand, the numerical results of this study indicate that the confinement and the aggregate size significantly influence the triaxial compressive strength and that a higher-confinement condition corresponds to a more significant aggregate-size effect. Thus, it is necessary to modify the KCC strength criterion to take the aggregate gradation into account.

(7) $$\begin{array}{c} \left\{\begin{array}{c} \sigma_{y}=[a_{0y}+p/\left(a_{1y}+a_{2y}p\right)]{\left(d_{max}/d_{0}\right)}^{\gamma_{y}}\\ \sigma_{m}=[a_{0m}+p/\left(a_{1m}+a_{2m}p\right)]{\left(d_{max}/d_{0}\right)}^{\gamma_{m}}\\ \sigma_{r}=[p/\left(a_{1r}+a_{2r}p\right)]{\left(d_{max}/d_{0}\right)}^{\gamma_{r}} \end{array}\right. \end{array}$$

where $d_{max}$ and $d_{0}$ denote the maximum aggregate size and the reference aggregate size, respectively. $\gamma_{y}$, $\gamma_{m}$, and $\gamma_{r}$ are the parameters related to aggregate gradation for the strength surfaces. In this study, $d_{0}$ is set to 20 mm.

In this study, the modified strength criterion of the KCC model is illustrated in Equation (7) by introducing the aggregate gradation term, where the yield strength surface is predicted with $\sigma_{y}=0.45\sigma_{m}$ and the residual strength surface is parallel to the maximum strength surface, as per the recommendations of Malvar et al. [24] and Kong et al. [25]. Then, based on the testing data and numerical results in this study, model parameters were provided for the modified strength surfaces herein through the least squares method. Table 3 lists the parameters of three strength surfaces, and the fitting result is also compared with the data in Figure 8. It is obvious that the proposed KCC strength criterion can effectively describe the aggregate-gradation effect of hydraulic concrete.

### 4.3. Modification of Strength Criterion for the HJC Model 

The HJC model assumes that the material behaves elastically before damage and that material damage accumulates under the following loading until total failure. After that, the material remains in a residual state. Generally, the strength criterion of HJC model can be illustrated by Equation (8), as follows:

(8) $$\begin{array}{c} \sigma^{*}=\left[A\left(1-D\right)+Bp^{*N}\right](1+C\;\ln\dot{\varepsilon }/{\dot{\varepsilon }}_{0}) \end{array}$$

(9) $$\begin{array}{c} \sigma^{*}=\left[A\left(1-D\right)+Bp^{*N}\right](1+C\;\ln\dot{\varepsilon }/{\dot{\varepsilon }}_{0}){\left(d_{max}/d_{0}\right)}^{\gamma } \end{array}$$

where *D* denotes the damage scalar, which varies from 0.0 to 1.0. The normalized strength and normalized pressure are defined as $\sigma^{*}=\sigma /f_{c}$=$\sqrt{3J_{2}}/f_{c}$ and $p^{*}=p/f_{c}$, respectively. The equivalent plastic strain rate is defined as ${\dot{\varepsilon }}^{*}=\dot{\varepsilon }/{\dot{\varepsilon }}_{0}$, and the reference strain rate ${\dot{\varepsilon }}_{0}$ equals 1 s^−1^. In addition, *A*, *B*, *N*, and *C* are the constant parameters.

To reflect the concrete aggregate-gradation effect, the HJC strength criterion is modified as Equation (9) by the introduction of the aggregate gradation term. It is noted that the strain-rate effect is not apparent when the strain rate is less than reference strain rate (${\dot{\varepsilon }}_{0}=1s^{-1}$) [10]. In this study, the triaxial compressive tests were conducted under the quasi-static condition and the loading rate was set to 1.0 × 10^−5^ s^−1^. Thus, the modified HJC strength criterion ignores the strain rate term (i.e., setting $(1+C\;\ln\dot{\varepsilon }/{\dot{\varepsilon }}_{0})\approx 1.0$) for simplification. On the other hand, Holmquist et al. set the normalized cohesive strength *A* to 0.79 by assuming the cohesive strength to be ${0.75f}_{c}$ [26]. However, Malvar et al. recommended 0.30 as a more reasonable *A* for concrete material based on the analysis of numerous experimental data, and this value was also used in this study [24]. Using the current experimental data and numerical results herein, the model parameters can be obtained for the modified strength criterion in the HJC model.

**Figure 9:**
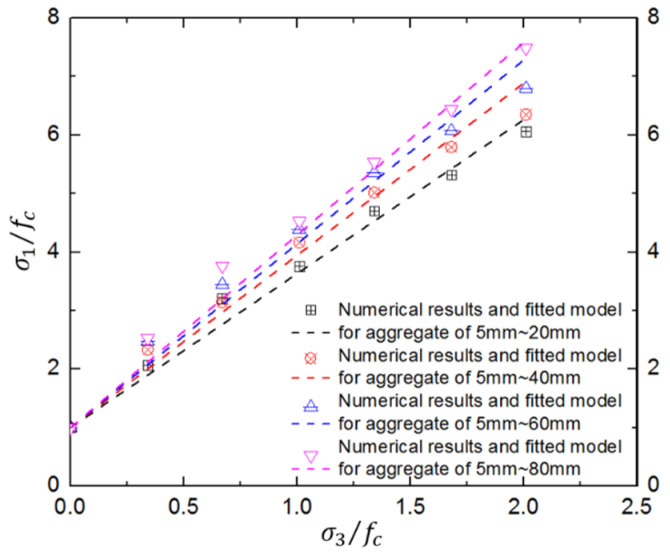
Determination of the modified HJC strength criterion for fully graded concrete.

There is no yield surface in the HJC model, and the parameters of *B*, *N*, and γ can be obtained through least squares fitting method, as shown in Table 4. Figure 9 compares the modified strength criterion with available triaxial compressive data for concrete with different aggregate gradations. It was found that the modified failure surface increases with the maximum aggregate size, in contrast to the original HJC failure surface. Although the failure surfaces are relatively similar when the confinement is small, a more obvious underestimate of the strength occurs when the maximum aggregate size gets larger at a high confinement.

### 4.4. Modification of thenStrength Criterion for RHT Model

The RHT model integrates the strain hardening and the third invariant dependence [27], in which the failure surface $F_{fail}$ is defined as a function of the normalized pressure $p^{*}$, Lode angle θ, and strain rate $\dot{\varepsilon }$, as expressed by Equation (10), below:

(10) $$\begin{array}{c} \left\{\begin{array}{c} F_{fail}\left(p^{*},\theta ,\dot{\varepsilon }\right)=Y_{c}\left(p^{*}\right)r_{3}\left(\theta \right)F_{rate}\left(\dot{\varepsilon }\right)\\ Y_{c}\left(p^{*}\right)=f_{c}\left[A_{c}{\left(p^{*}-f_{t}^{*}F_{rate}\left(\dot{\varepsilon }\right)\right)}^{N_{c}}\right] \end{array}\right. \end{array}$$

where $Y_{c}\left(p^{*}\right)$ is the compressive meridian of failure surface; $r_{3}\left(\theta \right)$ is a scalar function of Lode angle θ and the tensile-to-compressive meridian ratio ψ, as illustrated by Equation (11); $A_{c}$ and $N_{c}$ are two model parameters; $f_{t}^{*}=f_{t}/f_{c}$ and $f_{t}$ is the uniaxial tensile strength; and $F_{rate}\left(\dot{\varepsilon }\right)$ denotes the strain–rate effect. The formula is as follows:

(11) $$\begin{array}{c} r_{3}\left(\theta \right)=\frac{r}{r_{c}}=\frac{2\left(1-\psi^{2}\right)\cos\theta +\left(2\psi -1\right)\sqrt{4\left(1-\psi^{2}\right)\cos^{2}\theta +5\psi^{2}-4\psi }}{4\left(1-\psi^{2}\right)\cos^{2}\theta +{\left(1-2\psi \right)}^{2}} \end{array}$$

where $\psi =r_{t}/r_{c}$ represents the meridian ratio of tensile to compressive strength, which is a function of pressure. In the RHT model, AUTODYN defines the tensile-to-compressive meridian ratio to be linearly dependent on the hydrostatic pressure by AUTODYN, while a piecewise linear definition is used in the KCC model. Tu and Lu discussed the meridian ratio in detail and recommended a linear relationship defined as $\psi \left(p\right)=0.60+0.05p^{*}$ for a more accurate and simple description [28].

The yield surface $Y_{y}\left(p^{*}\right)$ was defined by the maximum failure surface and cap function $F_{cap}$, as expressed by Equations (12) and (13), as follows:

(12) $$\begin{array}{c} Y_{y}\left(p^{*}\right)=F_{cap}Y_{c}\left(p^{*}\right) \end{array}$$

(13) $$\begin{array}{c} F_{cap}=\left\{\begin{array}{cc} 1 & p\leq p_{u}\\ {\left[1-({\frac{p-p_{u}}{p_{0}-p_{u}})}^{2}\right]}^{1/2} & p_{u}<p<p_{0}\\ 0 & p_{0}\leq p \end{array}\right. \end{array}$$

where $p_{u}$ is the crushing pressure and $p_{0}$ is the upper limit of the cap.

In order to reflect the aggregate-gradation effect in the RHT strength criterion, the compressive meridian was modified to yield Equation (14). Thus, the contribution of aggregate gradation to the strength criterion of hydraulic concrete could be integrated into the failure surface and the yield surface. In addition, as discussed in Section 4.3, the strain-rate sensitivity was ignored in the compressive meridian herein due to the quasi-static loading condition (i.e., $F_{rate}\left(\dot{\varepsilon }\right)=1.0$). On the other hand, for concrete, previous studies suggested $f_{t}^{*}$ to be within 0.07 to 0.13 [27,29], while LS-DYNA defaults the value to be 0.10 [30]. In this study, $f_{t}^{*}$ was also assumed to be 0.10 for fully graded concrete, as follows:

(14) $$\begin{array}{c} Y_{c}\left(p^{*}\right)=f_{c}A_{c}{\left(p^{*}-f_{t}^{*}F_{rate}\left(\dot{\varepsilon }\right)\right)}^{N_{c}}{\left(d_{max}/d_{0}\right)}^{\gamma_{c}} \end{array}$$

where $\gamma_{c}$ is the parameter related to aggregate gradation in the RHT strength criterion.

**Figure 10:**
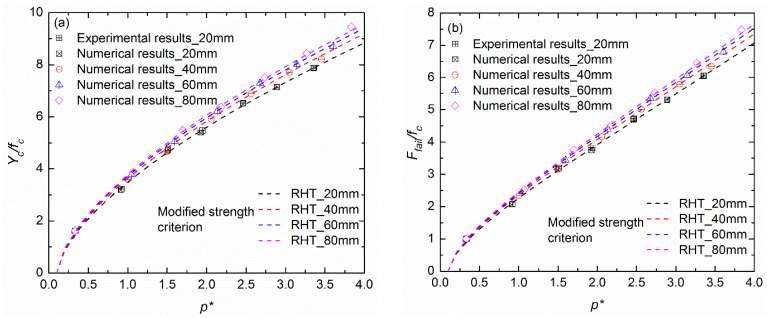
Determinations of the modified strength criterion in RHT model for fully graded concrete: (**a**) normalized compressive meridian; (**b**) normalized failure surface.

Figure 10 shows the normalized compressive meridian and the normalized failure surface compared with the available experimental data and numerical results for concrete obtained in this study. The model parameters for the modified RHT strength criterion were obtained by the least squares method with the available data, as listed in Table 5. It is apparent in Figure 10 that the modified RHT strength criterion can effectively describe the aggregate-gradation effect, avoiding the strength underestimation in the condition of large aggregates used in fully graded concrete.

## 5. Conclusions

Because of differences in aggregate gradation between hydraulic concrete and normal concrete, the existing strength criterion cannot be directly employed for fully graded hydraulic concrete. New strength criteria need to be derived by taking the aggregate gradation into consideration, and, typically, grade-three or grade-four concrete is widely applied in the concrete mix used in hydraulic engineering. However, this research has been limited by the size of the experimental equipment. Thus, this study focuses on the aggregate-gradation effect on the triaxial compressive behavior and tries to modify the widely used strength surfaces in concrete constitutive models to take the aggregate gradation into consideration. The following conclusions can be drawn:

(1) The available testing data and three-dimensional mesoscopic numerical results indicate a significant aggregate-gradation effect, where both the normalized axial compressive strength and the corresponding axial strain increase with increasing confinement. Moreover, at low confining pressures, the influence of aggregate gradation on concrete compressive strength is relatively limited. Concrete strength will increase with increasing aggregate size, and this aggregate-gradation effect is particularly significant under high confining pressures.
(2) The mechanism of the aggregate-gradation effect may be that smaller coarse aggregates in concrete lead to more ITZs, resulting in strength attenuation, which is amplified by the confinement effect. For example, when the maximum aggregate size increases from 20 mm to 80 mm, the increase of normalized peak strength reaches 23.49% at a confinement ratio of 2.01. Thus, the aggregate-gradation effect of fully graded concrete is especially significant at high confinement levels, especially when the concrete is subjected to high confinements such as extreme blast and impact loads.
(3) To emphasize the aggregate-gradation effect in the extreme-blast and impact conditions, the strength criteria in concrete dynamic constitutive models are further discussed. In this study, a modified strength criterion is derived by introducing the maximum aggregate factor to better represent fully graded concrete material, and the corresponding model parameters are fitted based on the available testing data and three-dimensional mesoscopic numerical results.

## Figures and Tables

**Table 1:** KCC constitutive parameters of mesoscopic components in concrete.

Parameters	Unit	Coarse Aggregate	Mortar	ITZ
Density ρ	kg·m^−3^	2600	2000	1800
Shear modulus G	GPa	18.50	10.63	8.65
Poisson’s ratio ν		0.20	0.167	0.167
$\mathrm{Tensile}\;\mathrm{strength}\;f_{t}$	MPa	5.20	2.10	1.80
$\mathrm{Compressive}\;\mathrm{strength}\;f_{c}$	MPa	50	15	10

**Table 2:** Peak strength of concrete under various confinements.

$\sigma_{3}/f_{c}$	$\sigma_{1}$ (MPa)				
	Laboratory Test 5 mm~20 mm	Mesoscopic Simulation			
		5 mm~20 mm	5 mm~40 mm	5 mm~60 mm	5 mm~80 mm
0	14.9	14.8	13.9	13.3	12.8
0.34	31.1	30.5	32.4	32.8	32.3
0.67	47.3	47.4	43.7	45.8	48.1
1.01	56.9	55.6	57.8	58.3	57.9
1.34	70.3	69.6	69.7	71.2	70.8
1.68	79.1	78.7	80.5	80.7	82.4
2.01	90.1	89.7	88.2	90.4	95.8

**Table 3:** Parameters for proposed KCC strength criterion.

Maximum Strength Surface		Yield Strength Surface		Residual Strength Surface		Aggregate Gradation	
$a_{0m}$	$0.340f_{c}$	$a_{0y}$	$0.153f_{c}$	$a_{0f}$	0	$\gamma_{m}$	0.0595
$a_{1m}$	0.515	$a_{1y}$	1.145	$a_{1f}$	0.515	$\gamma_{y}$	0.0595
$a_{2m}$	$0.0218/f_{c}$	$a_{2y}$	$0.0484/f_{c}$	$a_{2f}$	$0.0218/f_{c}$	$\gamma_{r}$	0.0595
						$d_{0}$	20 mm

**Table 4:** Parameters of the modified HJC strength criterion for fully graded concrete.

Strength Surface			Aggregate Gradation	
A	B	N	γ	$d_{0}$
0.30	1.514	0.7549	0.1182	20 mm

**Table 5:** Material parameters of the modified RHT strength criterion.

Strength Surface Parameters		Aggregate Gradation Parameters	
$A_{c}$	$N_{c}$	$\gamma_{c}$	$d_{0}$
3.729	0.634	0.0572	20 mm

## Data Availability

The original contributions presented in the study are included in the article, further inquiries can be directed to the corresponding author.
